# Synergy effect of talent policies on corporate innovation—Evidence from China

**DOI:** 10.3389/fpsyg.2022.1069776

**Published:** 2023-01-18

**Authors:** Qiuling Chen, Ting Sun, Tianchi Wang

**Affiliations:** School of Economics, Shanghai University, Shanghai, China

**Keywords:** talent policy, policy mixes, corporate innovation, talent gathering, threshold effect

## Abstract

The talent policy is a powerful tool for the government to implement and the talent is the key resources attributed to corporate innovation. Different types of talent policy instruments need to be synergistically combined to promote corporate innovation. By using the sample of China’s listed companies during the period 2007–2020, this paper applies the multidimensional fixed-effect OLS method to explore the impact of different types of talent policies and talent policy mixes on corporate innovation, and adopts threshold regression model to detect the threshold effect of talent gathering in the framework of government-enterprise interaction. The results are shown as follows: The supply-side talent policy (STP), demand-side talent policy (DTP), and environmental-side talent policy (ETP) all positively affect corporate innovation. Talent policy mixes have a significant synergy on corporate innovation. And the effect of STP- DTP-ETP mixes is greater than that of any two types of talent policy mixes. Talent gathering has a threshold effect on the relationship between STP-DTP-ETP mixes and corporate innovation. Our study provides empirical evidence of the positive impact of different types of talent policy and their mixes on corporate innovation and enriches the literature related to talent gathering.

## 1. Introduction

Firm-specific human capital has been viewed as critical to fostering innovation and greater productivity. According to the resource-based theory, human capital is viewed as a key resource, and performance differences across firms can be also attributed to the variations in the resources of the firm (Custódio et al., 2019). A growing number of articles also demonstrated how businesses may use human resources to encourage innovation that improves organizational sustainability in social, environmental, and commercial contexts (e.g., Centobelli et al., 2017; Lopes et al., 2017). Globally, decision-makers are becoming more aware of the crucial role that human capital serves in the economic development of the nation, which is fueling the “war for talent” (Kapur and McHale, 2005). The US has established a new immigration policy based on family reunification and skilled labor attraction (Chand and Tung, 2019). Compared to other countries, the Chinese government has considerable authority in resource allocation because of the distinct culture and institution in China (Okhmatovskiy, 2010). The Chinese government has begun to highlight the importance of talent in moving Chinese economic development mode from quantity to quality, and has advocated a variety of policy subsidies to recruit and retain talent (Yang and Pan, 2020). Indeed, the talent policies can help firms to concentrate their advantages, break through the essential core technologies of the industry, so as to stimulate the vitality of corporate innovation.

From the perspective of policy content, some talent policies emphasize direct incentives for high-level talent, while some policies strengthen the support for enterprise talent research, such as setting up special financial subsidy funds or projects. The previous studies focus on one type of talent policy (e.g., Toma and Villares-Varela, 2019; Xu et al., 2022), but it was insufficient to study different types of talent policies simultaneously. In fact, talent policies include a series of systematic, regular and systematic practices that can generate long-term returns for enterprises. Specifically, talent policies provide support for talent from a multi-dimensional perspective, including attract, retain, and cultivate talent. According to Rothwell and Zegveld (1981), the talent policy instruments can be divided into the supply-side talent policy (STP), demand-side talent policy (DTP), and environmental-side talent policy (ETP). Therefore, it is necessary to study different types of talent policies and investigate the influence of talent policies on corporate innovation.

Furthermore, Weber and Rohracher (2012) argued that combining various types of policies might better solve conventional market and transformation system failure, therefore contributing to the sustainable growth of both society and the economy. Reichardt et al. (2016) believed that policy mixes are a method for managing and improving policy effectiveness. That is, the effect of a unitary kind of policy on enterprise innovation ignores the interaction between different types of policies. Therefore, it is necessary to synergistically combine different policy instruments in the research of enterprise innovation. However, there is still less empirical research on the relationship between different talent policy mixes and corporate innovation. In addition, talent gathering enhances the interaction among talent and forms closer inter-organizational cooperation (Liu et al., 2022). Unfortunately, the role of talent gathering in government-enterprise interaction has not been fully discussed in previous studies. When the level of talent gathering is higher, it enables companies to benefit more from heterogeneous resources, including different types of talent subsidies. Therefore, when studying the impact of talent policies mixes on corporate innovation, the nonlinear influence of talent gathering should be considered.

Hence, we pose three research questions: (1) Does STP, DTP, and ETP all have a positive effect on corporate innovation? (2) Do different types of talent policy mixes contribute to the improvement of corporate innovation performance? (3) Does talent gathering have a nonlinear effect on the influence of STP-DTP-ETP mixes on corporate innovation? Based on the listed companies of China A-shares from 2007 to 2020, we apply the multidimensional fixed-effect OLS and threshold regression models to explore the impact of different types of talent policies and talent policy mixes on corporate innovation, and to detect the threshold effect of talent gathering between supply-side, demand-side, and environmental-side talent policy synergies and corporate innovation.

The possible contributions of this study are as follows: First, existing studies have focused on a certain type of talent policy, but research on the effect of different talent types has been scarce. This paper classifies talent policy instruments from the demand, supply and environmental sides. Based on the large sample including 33,441 observations, we uncover the positive effect of the STP, DTP, and ETP mixes on corporate innovation. Second, we explore the synergistic effect of different talent policy mixes on corporate innovation and further compare the effects of different types of talent policy mixes. We find that the mix talent policies are beneficial to corporate innovation. And the effect of STP-DTP-ETP mixes is better than two types of talent policy mixes, which illustrates that the more various types of policy synergy, the better to enterprise innovation. Finally, this paper contributes to extant literature by introducing talent gathering as a threshold variable. We reveal the threshold effect of talent gathering between the three talent policy mixes and corporate innovation. This type of threshold variable is relatively rare in the interaction between government and enterprises.

The remainder of this study is arranged as follows: Section 2 lays out the theoretical framework and proposes hypotheses. Section 3 constructs the methodology and introduces the data. Section 4 displays the results. Section 5 refers to discussion. The final section presents the conclusions and implications.

## 2. Literature review and hypothesis development

### 2.1. Theoretical basis and talent policy instrument

Talent policy is becoming an effective tool for authorities to support corporate innovation in both developing and developed countries (Huang et al., 2004; Reiner et al., 2017). According to the externality theory, policies should be developed to encourage investment in company innovation (Li Q. et al., 2021). Practically speaking, nearly all governments intervened to correct the positive externalities of innovation caused by market failure (Szücs, 2020), thereby hoping to promote enterprises’ innovation ability. Therefore, the government has introduced talent policies to support the development of enterprises. In particular, Liu and Tian (2021) found that talent policies could motivate firms to innovate, enhance their business credibility, and increase R&D investment. However, the previous study focuses on one type of talent policy (e.g., Toma and Villares-Varela, 2019; Xu et al., 2022), but it was insufficient to study different types of talent policies at the same time. Prior scholars deemed that research focused on a single policy might lead to potential bias (Guerzoni and Raiteri, 2015; Busom et al., 2017). Thus, scholars have made various classifications of policies. The most representative classification is proposed by Rothwell and Zegveld (1981) who classified the policy instruments into supply-side policy, demand-side policy and environmental-side policy. Therefore, we divided the talent policies into supply-side talent policy, demand-side talent policy, and environmental-side talent policy. STP means that the government provides support for technical innovation by applying the policy instruments which include talent training and education, talent infrastructure construction, and talent funding. DTP refers to the government developing and stabilizing the talent market through talent introduction, industry-university-research cooperation, incentive subsidies. ETP reflects the influence of policies on talent development, i.e., tax incentives, intellectual property protection. The government sustains a favorable development environment for talents with the help of financial and monetary, taxation systems, regulations, and control policies.

### 2.2. Talent policies and corporate innovation

According to the classification of STP, the talent training and education policies are the “Reservoir” of corporate talent and continuously cultivate talent, promote the quality of talent and increase the stock of human capital of the company. And the human resources are the key factor in corporate innovative performance. Moreover, the talent infrastructure establishes a platform to promote the flow of information among industries. It also encourages talent to full play with their abilities to improve corporate innovation. Furthermore, talent funding has a signal transmission function. That is, companies supported by talent policies send signals to stakeholders that the company “has talent strengths and development capabilities” and is “trustworthy” (Li et al., 2019). Consequently, the concerns from the external investors about the creditworthiness of the firms and the information asymmetry between the companies and their stakeholders can be decreased, and positive evaluations and support are gained from stakeholders.

The DTP directly drives the demand for enterprise innovation on the demand side, which helps to form an important connection between innovation output and the market, and then encourages enterprises to innovate (Edler et al., 2012). Previous research demonstrated that the ability of innovation policies to motivate the employees of a firm determined the effectiveness in increasing the output of innovation outcomes of the firm (Koroglu and Eceral, 2015). The talent introduction is the most important demand-side innovation policy in China, which can help enterprises attract all kinds of outstanding talent, resulting in stimulating enterprise innovation. Favorable payment and subsidies is a crucial motivator to attract talented workers (Rynes et al., 2004). Incentive subsidies also inject funds into enterprise innovation, stimulate vitality of enterprise innovation, and reduce innovation uncertainty.

In contrast to STP and DTP which directly support corporate innovation, the impact of ETP on enterprises is to provide a stable market environment for innovation, thereby avoiding the tendency of “rent-seeking behavior” from enterprises (Zhou, 2022). On the one hand, the ETP gives enough security, so that the talent has the motivation and willingness to actively participate in innovation work. Indeed, a good innovation atmosphere can encourage staff to freely discuss and test out novel concepts and methods (Mokhber et al., 2018), prompt them to generate innovation intentions, and then transform the intentions into practical actions (Wang et al., 2017). On the other hand, the ETP reduces the investment cost of enterprise innovation through tax subsidies and tax breaks (Dai and Chapman, 2022), so as to encourage enterprises to employ more skilled talent. In addition, since talent tax incentives have a series of identification criteria which may prompt firms to actively cater to external requirements and absorb the right talent, thus improving the capabilities of corporate innovation. Consequently, the following hypothesis is put forward.

*H1*: The STP, DTP, and ETP can positively affect corporate innovation, respectively.

### 2.3. Talent policy mixes and corporate innovation

In the context of close ties between government and society, the application of a single policy tool is generally differentiated and time-sensitive, and it is challenging to address all issues individually. Pang et al. (2020) believed that the study of a single policy could disregard the contribution of other policies, and produce biased and incomplete results. Different types of policy mixes are often needed, as one type of policy tool cannot address all known flaws, bottlenecks, or threats (Schmidt and Sewerin, 2019). In other word, policy goals can be best met when numerous policies are appropriately nested and coordinated. Since companies frequently get numerous innovation policy instruments at the same time, the interactions among a portfolio of these instruments may have a significant impact on the impact of innovation policy that is observed (Martin, 2016). Policy synergy is advantageous for increasing the effectiveness of policies and achieving Pareto optimum (Iglesias et al., 2011). Therefore, different types of talent policy instruments should be synergistically combined. Policy mix can provide support for talent development in a comprehensive manner. Specifically, demand-side talent policies drive innovation demand, supply-side talent policies provide innovation support, and environmental-side talent policies improve the talent innovation environment. The combination of talent policy instruments promotes each other so that the effect of talent policy implementation can be expanded. Hence, this paper argues that different talent policy mixes have synergistic effects on corporate innovation. The following hypothesis is proposed.

*H2*: Talent policy mixes have significant synergy effects on corporate innovation.

### 2.4. The threshold effect of talent gathering

Talent gathering plays an important role in the relationship between STP-DTP-ETP mixes and corporate innovation. First, the absorptive capacity theory states that the stronger an enterprise’s absorptive ability, the greater it can utilize external resources (Tsai, 2001). The source of the enterprise’s absorption capacity is its employees (Minbaeva et al., 2003). Because of the talent gathering, a company can better use the different types of talent policies, thus realizing complementary advantages and improving innovation performance. Second, talent gathering can maximize the advantages of talent groups and produce clustering effects such as information sharing, collective learning, and knowledge spillover (Howitt, 1999). And closeness to talent enhances the frequency of information exchange (Wang et al., 2020). More cooperation and information exchange are capable of making full use of different types of talent policies. Finally, when the talents gathering in enterprises reaches a certain level, the interaction between talent can be enhanced and a more favorable innovation atmosphere can be formed, which makes the talent policy mixes to generate greater effect in promoting enterprise innovation. Thus, this paper argues that the relationship between STP-DTP-ETP mixes and corporate innovation is not simply linear but may present some mutation phenomenon, i.e., threshold effect. In other words, the effect of STP-DTP-ETP mixes on corporate innovation may vary depending on the degree of talent gathering. We propose the following hypothesis based on the above reasoning.

*H3*: The STP-DTP-ETP mixes have a nonlinear effect on corporate innovation. When talent gathering is greater than the threshold value, the contribution of STP-DTP-ETP mixes to corporate innovation can be enhanced.

**Figure 1:**
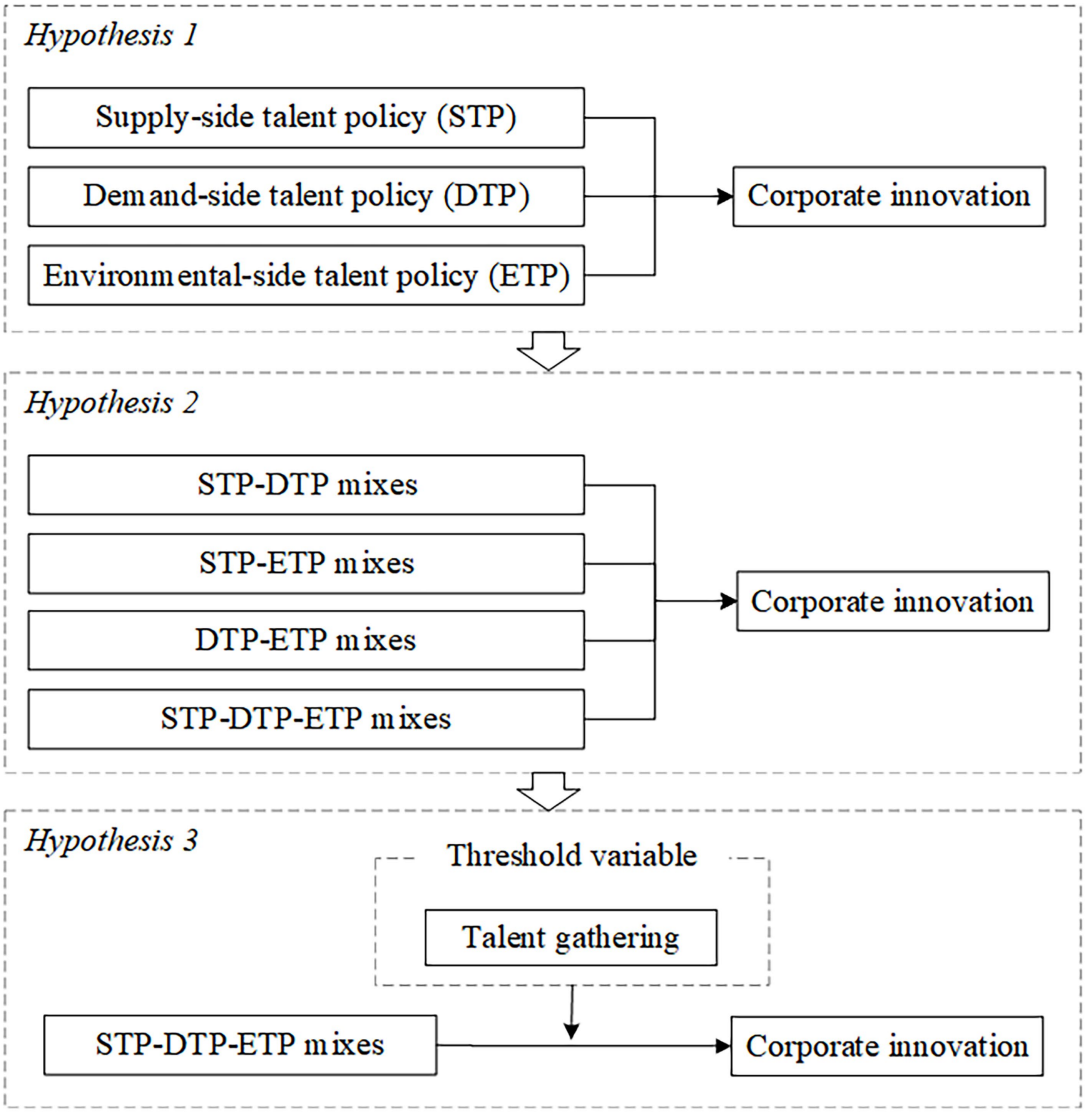
Process map.

The analysis presented above leads to Figure 1, which illustrates the process map.

## 3. Methodology

### 3.1. Empirical model specification

#### 3.1.1. Multidimensional fixed-effect OLS method

In contrast to the ordinary fixed-effect model, the multidimensional fixed-effect model can simultaneously include over two fixed effects and capture unobserved heterogeneity (e.g., industry and region) (Xu, 2018), and correctly calculate clustering robustness errors (Gormley and Matsa, 2014). Therefore, this study uses the multidimensional fixed-effect OLS method to examine the influence of different types of talent policies and their mixes on corporate innovation by controlling for region, industry, and year fixed effects. Specifically, we analyze the impact of different talent policies on innovation and detect the synergy effect of STP-DTP-ETP mixes on corporate innovation. In reference to Zhu and Xu (2003) and Wei and Cao (2019), the policy mixes are measured by their interactions. The models are constructed as follows:


(1) $$\begin{array}{l} {\mathrm{CI}}_{i,t}=\alpha_{0}+\alpha_{1}{\mathrm{TP}}_{i,t}+\theta_{i}X_{i,t}+\delta_{i}+\\ \;\;\;\;\;\;\;\;\;\;\;\;\;\;\;\;\mu_{i}+\pi_{i}+\varepsilon_{i,t}\;\left(\mathrm{TP}\in \mathrm{STP,DTP,ETP}\right) \end{array}$$

(2) $$\begin{array}{l} {\mathrm{CI}}_{i,t}=\alpha_{0}+\alpha_{1}{\mathrm{STP}}_{i,t}+\alpha_{2}{\mathrm{DTP}}_{i,t}+\alpha_{3}{\mathrm{STP}}_{i,t}*\\ \;\;\;\;\;\;\;\;\;\;\;\;\;\;\;\;{\mathrm{DTP}}_{i,t}+\theta_{i}X_{i,t}+\delta_{i}+\mu_{i}+\pi_{i}+\varepsilon_{i,t} \end{array}$$

(3) $$\begin{array}{l} {\mathrm{CI}}_{i,t}=\alpha_{0}+\alpha_{1}{\mathrm{STP}}_{i,t}+\alpha_{2}{\mathrm{ETP}}_{i,t}+\alpha_{3}{\mathrm{STP}}_{i,t}*\\ \;\;\;\;\;\;\;\;\;\;\;\;\;\;\;\;\;{\mathrm{ETP}}_{i,t}+\theta_{i}X_{i,t}+\delta_{i}+\mu_{i}+\pi_{i}+\varepsilon_{i,t} \end{array}$$

(4) $$\begin{array}{l} {\mathrm{CI}}_{i,t}=\alpha_{0}+\alpha_{1}{\mathrm{DTP}}_{i,t}+\alpha_{2}{\mathrm{ETP}}_{i,t}+\alpha_{3}{\mathrm{DTP}}_{i,t}*\\ \;\;\;\;\;\;\;\;\;\;\;\;\;\;\;\;{\mathrm{ETP}}_{i,t}+\theta_{i}X_{i,t}+\delta_{i}+\mu_{i}+\pi_{i}+\varepsilon_{i,t} \end{array}$$

(5) $$\begin{array}{l} {\mathrm{CI}}_{i,t}=\alpha_{0}+\alpha_{1}{\mathrm{STP}}_{i,t}+\alpha_{2}{\mathrm{DTP}}_{i,t}+\alpha_{3}{\mathrm{ETP}}_{i,t}+\\ \;\;\;\;\;\;\;\;\;\;\;\;\;\;\;\;\alpha_{4}{\mathrm{STP}}_{i,t}*{\mathrm{DTP}}_{i,t}*{\mathrm{ETP}}_{i,t}+\theta_{i}X_{i,t}+\\ \;\;\;\;\;\;\;\;\;\;\;\;\;\;\;\;\delta_{i}+\mu_{i}+\pi_{i}+\varepsilon_{i,t} \end{array}$$

where $\;{\mathrm{CI}}_{i,t}$ represents the corporate innovation of firm *i* in year *t.* ${\mathrm{TP}}_{i,t}$ is represented as the talent policies of firm *i* in year *t*, including STP, DTP, and ETP. $X_{i,t}$ refers to the control variables. $\delta_{i}$ , $\mu_{i}$ , and $\pi_{i}$ are region, industry, and year fixed effects, respectively. $\varepsilon_{i,t}$ stands for the robust standard error. Model (1) tests H1 and Models (2)–(5) test H2.

#### 3.1.2. Threshold regression model

To explore the threshold effect of talent gathering, this paper introduces the panel threshold regression model proposed by Hansen (1999) into the research of the nonlinear effect of STP-DTP-ETP mixes on corporate innovation. This paper constructs a multi-threshold regression model.

(6) $$\begin{array}{l} {\mathrm{CI}}_{i,t}=\gamma_{0}+\gamma_{1}\mathrm{STP}*\mathrm{DTP}*{\mathrm{ETP}}_{i,t}*\;D\left({\mathrm{TG}}_{i,t}\leq \eta_{1}\right)+\;\\ \;\;\;\;\;\;\;\;\;\;\;\;\;\;\;\;\gamma_{2}\mathrm{STP}*\mathrm{DTP}*{\mathrm{ETP}}_{i,t}*\;D\left(\eta_{1}<{\mathrm{TG}}_{i,t}\leq \eta_{2}\right)+\cdots +\\ \;\;\;\;\;\;\;\;\;\;\;\;\;\;\;\;\gamma_{n}\mathrm{STP}*\;\;\mathrm{DTP}*{\mathrm{ETP}}_{i,t}*D\left(\eta_{n-1}<{\mathrm{TG}}_{i,t}\leq \eta_{n}\right)+\\ \;\;\;\;\;\;\;\;\;\;\;\;\;\;\;\;\rho_{i}X_{i,t}+\varepsilon_{i,t}\cdots \left(n>2\right) \end{array}$$

${\mathrm{TG}}_{i,t}$ reflects the talent gathering of firm *i* in year t. $\eta_{1}$ , $\eta_{2}$ ,… $\eta_{n}$ mean n different thresholds. Other variables are the same as in Section 3.3.1.

### 3.2. Data source

Our sample is comprised of listed firms on the Shanghai Stock Exchange (SHSE) and Shenzhen Stock Exchange (SZSE) during the period 2007–2020. To avoid survivorship bias, our sample includes all companies for which the relevant data are available even if they are currently extinct. Due to the particularity of the financial data, finance and insurance companies are excluded (Li K. et al., 2021). Observations with missing variables are then removed. According to the above criteria, 33,441 observations from 3,485 companies are finally retained. The patent data are obtained from the CNRDS system, and CSMAR system and the Market Ability Index Marketization Index of China.

### 3.3. Variable description

#### 3.3.1. Dependent variable

Patent is considered a necessary condition for innovative companies to maintain their technological competitiveness by having intellectual property rights (Ai and Zhang, 2021). Based on the existing study by Beladi et al. (2022), the number of patents granted should be a reliable and valid measure of corporate innovation. This paper thereby uses total number of patents granted as the indicator of corporate innovation (Zhang L. et al., 2022). Furthermore, the number of patents applied is also an important form to represent corporate innovation (Hou et al., 2017). To make the study more comprehensive, we take the number of patents applied into consideration in the robustness test part (i.e., Section 4.3.2).

#### 3.3.2. Independent variables

The independent variables include supply-side talent policy (STP), demand-side talent policy (DTP), and environmental-side talent policy (ETP) and their mixes. This paper uses the text search method to construct three types of talent policy instruments. The advantage of this method is that the data are valid and rigor because they are directly come from the companies. Referring to Shao and Chen (2022), we construct the talent policy indicator based on the China Stock Market and Accounting Research Database (CSMAR) which is a specializing database in Chinese corporate research. Its subsidiary database, the “sub-data of government subsidies,” discloses explicit information on government subsidies of all A-share listed companies, including “project name,” “project profile,” and the corresponding “subsidy amount.” Based on the textual features of the subsidies, we manually select the STP, DTP, and ETP by searching information with the keywords as shown in Table 1. Simultaneously, we merge the data of talent subsidies received by company to calculate the different types of talent policy indicators for each firm per year.

**Table 1:** The text search keywords of different talent policy.

Talent policies	Keywords
Supply-side talent policy	Selection, training, workstation, housing, medical, household registration, social insurance, talent incentives, talent founding, talent subsidies, social insurance, medical
Demand-side talent policy	Introduction, industry-academia-research, overseas, international exchange, assessment, evaluation, supervision, regulation, school-enterprise cooperation
Environmental-side talent policy	Talent tax relief, talent tax incentives, tax subsidies, intellectual property regulations, equity incentives, work allowances, loan guarantee

#### 3.3.3. Threshold variable

Referring to the study of Liu and Tian (2021), this paper uses the number of employees with master degree or above as the indicator for talent gathering in firms.

#### 3.3.4. Control variables

The control variables are as follows: Firm size, Liquidity ratio, Return on assets, Liability on asset ratio, Shareholding ratio of major shareholders, Political affiliation, and Market level. Firm size is represented by the natural logarithm of the total assets. Liquidity ratio is the current assets divided by the current liabilities (Yuan and Wen, 2018). Return on assets refers to the net profit divided by the total assets (Wen et al., 2022). Liability on asset ratio means total liabilities divided by total assets. Shareholding ratio of major shareholders can be calculated as the percentage of shares owned by the largest shareholder. Political affiliation stands for the natural logarithm of the number of business executives with political experience, and Market level can be reflected by the marketization index (Liu and Tian, 2021). Table 2 provides definitions of all variables used in our analysis and all continuous variables are winsorized at 1% at both tails to mitigate the undue influence of extreme values.

**Table 2:** Variable specification.

Category	Variable name	Variable symbol	Variable definition
Dependent variable	Corporate innovation	CI	The natural logarithm of the total number of patents granted plus one.
Independent variables	Supply-side talent policy	STP	The natural logarithm of funds for supply talent subsidy
	Demand-side talent policy	DTP	The natural logarithm of funds for demand talent subsidy
	Environmental-side talent policy	ETP	The natural logarithm of funds for environmental talent subsidy
Threshold variable	Talent Gathering	TG	The natural logarithm of the numbers of employees with master degree or above
Control Variables	Firm size	SIZE	The natural logarithm of the total assets
	Liquidity ratio	CR	Current assets divided by the current liabilities
	Return on assets	ROE	The net profit divided by the total assets
	Liability on asset ratio	LEV	Total liabilities divided by the total assets
	Shareholding ratio of major shareholders	TOP	The percentage of shares owned by the largest shareholder
	Political affiliation	PC	The natural logarithm of the number of business executives with political experience
	Market level	INDEX	Marketization index

## 4. Empirical results

### 4.1. Descriptive statistical analysis

The descriptive statistics are conducted for the main variables and the results are shown in Table 3. It can be seen that the average CI is 1.470, while the standard deviation of CI is 1.470, illustrating that there is a large difference in the level of innovation among different companies. The mean of STP, DTP, and ETP are 1.822 0.649, and 1.351, respectively, which means that STP and ETP are dominant in Chinese talent policies. And the TG has a mean of 2.244, and its standard deviation is 2.197. In addition, this paper calculates the value of VIF. The average VIF of 1.337 and the maximum VIF of 2.172 are much lower than 10, suggesting that multi-collinearity is not a severe concern in this study (Plank and Doblinger, 2018).

**Table 3:** Descriptive statistics of main variables and the value of VIF.

	*N*	MEAN	SD	MIN	MAX	VIF
CI	33,441	1.270	1.470	0.000	8.906	(1.337)
STP	33,441	1.822	4.473	0.000	15.179	1.209
DTP	33,441	0.649	2.739	0.000	17.504	1.135
ETP	33,441	1.351	3.643	0.000	17.226	1.121
TG	33,441	2.244	2.197	0.000	7.251	1.242
SIZE	33,441	22.023	1.316	19.368	26.086	1.457
CR	33,441	2.467	2.671	0.246	17.300	1.710
ROE	33,441	0.039	0.066	−0.289	0.210	1.216
LEV	33,441	0.434	0.214	0.051	0.975	2.172
TOP	33,441	35.117	15.061	8.730	75.100	1.095
PC	33,441	3.218	3.048	1.000	35.000	1.257
INDEX	33,441	8.276	1.995	−1.420	12.000	1.091

Table 4 is the Pearson Correlation matrix of the variables. The results show that there is a positive correlation between CI and (i) STP and (ii) DTP and (iii) ETP at the 1% significant level, indicating a significant positive correlation between different types of talent policy and corporate innovation. Meanwhile, most of the control variables pass the significance test at the 1 and 5% level, demonstrating that the select of control variables are reasonable.

**Table 4:** Correlation matrix of main variables.

	CI	STP	DTP	ETP	TG	SIZE	CR	ROE	LEV	TOP	PC	INDEX
CI	1											
STP	0.052^***^	1										
DTP	0.033^***^	0.331^***^	1									
ETP	0.060^***^	0.282^***^	0.184^***^	1								
TG	0.190^***^	0.120^***^	0.054^***^	0.094^***^	1							
SIZE	0.258^***^	−0.023^***^	−0.019^***^	−0.082^***^	0.364^***^	1						
CR	−0.053^***^	0.034^***^	0.025^***^	0.078^***^	−0.032^***^	−0.311^***^	1					
ROE	0.032^***^	0.023^***^	0.022^***^	0.019^***^	0.024^***^	−0.013^**^	0.224^***^	1				
LEV	0.042^***^	−0.081^***^	−0.046^***^	−0.118^***^	0.039^***^	0.418^***^	−0.641^***^	−0.369^***^	1			
TOP	0.026^***^	−0.061^***^	−0.040^***^	−0.055^***^	0.009	0.191^***^	−0.023^***^	0.141^***^	0.034^***^	1		
PC	0.092^***^	−0.077^***^	−0.052^***^	−0.087^***^	0.095^***^	0.387^***^	−0.182^***^	−0.041^***^	0.226^***^	0.211^***^	1	
INDEX	0.103^***^	0.062^***^	0.029^***^	0.020^***^	0.188^***^	0.045^***^	0.047^***^	0.050^***^	−0.122^***^	−0.023^***^	−0.151^***^	1

^*^, ^**^, and ^***^ indicate significance at the 10, 5, and 1% significance levels, respectively. The following tables are the same.

### 4.2. Regression analysis

In this section, we apply the multidimensional fixed-effect OLS method to test H1 and H2. The results are reported in Table 5. For Model 1, it can be seen that STP, ESP and DSP all have a significantly positive impact on corporate innovation. That is, any types of talent policies demonstrate incentive effects on innovation. This is in line with H1. Model 2 shows that the coefficient of STP-DTP mixes is significantly positive, indicating that STP-DTP mixes exert a synergy effect in the process of stimulating enterprise innovation. As presented in Model 3, the coefficient of STP-ETP mixes is significant and positive, illustrating that supply-side talent policies and environmental-side talent policies also exert a synergy effect during the process of stimulating enterprises’ innovation. The coefficient of the DTP-ETP mixes is significantly positive in Model 4, which indicates that demand-side talent policies and environmental-side talent policies do exert a certain synergy effect during the process of innovation. Model 5 shows that STP-DTP-ETP mixes significantly promote corporate innovation, indicating that the three policies interact with each other to exert a synergy effect in promoting innovation. H2 can be verified. Furthermore, the coefficient of STP-DTP-ETP mixes (0.112) is much larger than STP-DTP mixes (0.001), STP-ETP mixes (0.001), and DTP-ETP mixes (0.001), which illustrates that the synergy of STP-DTP-ETP mixes has a greater impact on firms’ innovation performance.

**Table 5:** Results of the multidimensional fixed-effect OLS model.

Variables	Single policy			Policy mixes			
	Model 1			Model 2	Model 3	Model 4	Model 5
STP	0.005^***^			0.004^***^	(0.002)		0.002^**^
	(0.001)			(0.001)	(0.012)		(0.001)
DTP		0.004^**^		(0.002)		(0.001)	(0.001)
		(0.002)		(0.002)		(0.002)	(0.002)
ETP			0.010^***^		0.005***	0.009***	0.008^***^
			(0.001)		(0.002)	(0.002)	(0.001)
STP*DTP				0.001^***^			
				(0.000)			
STP*ETP					0.001^***^		
					(0.000)		
DTP*ETP						0.001^**^	
						(0.000)	
STP*DTP*ETP							0.112^***^
							(0.041)
SIZE	0.199^***^	0.199^***^	0.200^***^	0.198^***^	0.199^***^	0.200^***^	0.199^***^
	(0.006)	(0.006)	(0.006)	(0.006)	(0.006)	(0.006)	(0.006)
CR	−0.007^***^	−0.007^***^	−0.007^***^	−0.007^***^	−0.007^***^	−0.007^***^	−0.007^***^
	(0.002)	(0.002)	(0.002)	(0.002)	(0.002)	(0.002)	(0.002)
ROE	0.231^***^	0.231^***^	0.234^***^	0.230^***^	0.229^***^	0.232^***^	0.231^***^
	(0.068)	(0.068)	(0.068)	(0.068)	(0.068)	(0.068)	(0.068)
LEV	−0.148^***^	−0.152^***^	−0.143^***^	−0.148^***^	−0.142^***^	−0.143^***^	−0.141^***^
	(0.028)	(0.028)	(0.028)	(0.028)	(0.028)	(0.028)	(0.028)
TOP	−0.001^***^	−0.001^***^	−0.001^***^	−0.001^***^	−0.001^***^	−0.001^***^	−0.001^***^
	(0.000)	(0.000)	(0.000)	(0.000)	(0.000)	(0.000)	(0.000)
PC	0.007^***^	0.007^***^	0.007^***^	0.007^***^	0.007^***^	0.007^***^	0.007^***^
	(0.002)	(0.002)	(0.002)	(0.002)	(0.002)	(0.002)	(0.002)
INEDX	(0.003)	(0.002)	(0.001)	−0.003	(0.001)	(0.001)	(0.001)
	(0.009)	(0.009)	(0.009)	(0.009)	(0.009)	(0.009)	(0.009)
Constant	−3.938^***^	−3.933^***^	−3.988^***^	−3.932^***^	−3.964^***^	−3.984^***^	−3.982^***^
	(0.148)	(0.148)	(0.147)	(0.148)	(0.147)	(0.147)	(0.147)
Year/Region/Industry	Yes	Yes	Yes	Yes	Yes	Yes	Yes
*N*	33,441	33,441	33,441	33,441	33,441	33,441	33,441
Adj-*R*^2^	0.143	0.142	0.144	0.143	0.145	0.144	0.144
*F*-value	179.316	172.858	183.721	144.004	150.116	147.975	136.634

The numbers in parentheses below the regression coefficients are robust standard errors. The following tables are the same.

**Figure 2:**
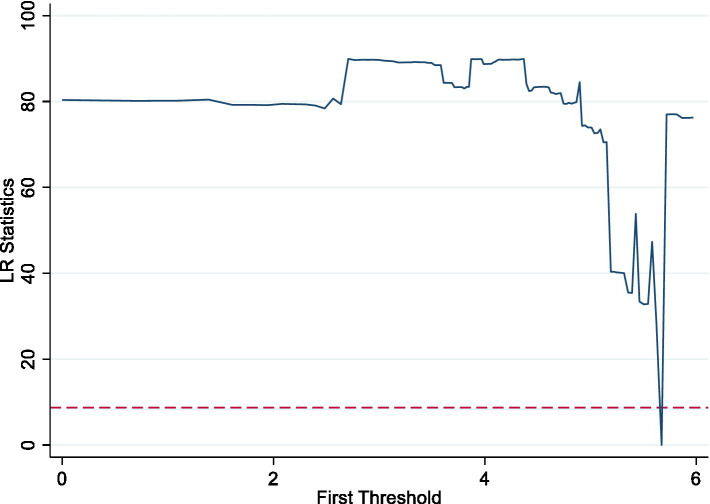
LR diagram of the threshold effect.

Confirming the existence and number of thresholds is important for the panel threshold regression model. Thus, we utilize Hansen’s (1999) Bootstrap “self-sampling” method and Stata 17.0 software to test the threshold effect. Table 6 presents the results. The results are shown in Table 6. Under the conditions of 300 self-sampling, the talent gathering passes the single threshold test at a 5% significance level and the corresponding *F* value is 81.070, while the double threshold is not significant. This demonstrates that by setting the talent gathering as the threshold variable, the STP-DTP-ETP mixes have a single threshold effect on corporate innovation, with a single threshold of 5.673 and a 95% confidence interval of [5.624, 5.720]. Figure 2 depicts the inflection point of the threshold value in the LR diagram.

**Table 6:** Results of the threshold effect test.

Threshold variable	Threshold type	Threshold value	*F*-value	*p*-value	Bootstrap times	Critical value of 95% significance levels
TG	Single threshold	5.673^**^	81.07	0.023	300	[5.624, 5.720]
	Double threshold	5.522	16.08	0.697	300	[5.329, 5.844]

Table 7 presents the result of single-threshold regression. In the range of low TG level (TG ≤ 5.673), STP-DTP-ETP mixes is positively related with CI at the 5% significant level. When TG is in the high level range (TG>5.673), the coefficient of STP-DTP-ETP mixes on CI is 0.097 which is almost double the former (0.048). This shows that with the continuous gathering of talent, the promotion effect of STP-DTP-ETP mixes on corporate innovation can be enhanced. This is consistent with H3.

**Table 7:** Results of the threshold effect model.

Variables	Coefficients	Robust standard errors	*T*-value	*p*-value
STP*DTP*ETP(TG ≤ 5.673)	0.048^**^	0.021	2.269	0.023
STP*DTP*ETP(TG > 5.673)	0.097^*^	0.052	1.860	0.063
SIZE	0.114^***^	0.005	22.418	0.000
CR	0.001	0.002	0.616	0.538
ROE	−0.255^***^	0.057	−4.458	0.000
LEV	0.027	0.031	0.862	0.389
TOP	−0.005^***^	0.000	−10.194	0.000
PC	0.015^***^	0.002	8.009	0.000
INDEX	0.029^***^	0.004	7.671	0.000
Constant	−2.313^***^	0.104	−22.246	0.000
*F*-value	161.684			

### 4.3. Robustness test

#### 4.3.1. Lag of independent variables

This paper uses the lagged value of independent variable to alleviate the endogeneity problem caused by reverse causality (Bellemare et al., 2017), the results are shown in Table 8. As shown in Model 1, the one-period lagged talent policies are all significantly correlated with corporate innovation at the 1% level of significance. The Models 2 to 5 show that the one-period lagged talent policy mixes are all significantly positive affect corporate innovation. Furthermore, the coefficient of the STP-DTP-ETP mixes (0.097) is larger than the coefficients of any two talent policy mixes. The results are relatively stable.

**Table 8:** Estimation for lagging one-period talent policies and their mixes.

Variables	Single policy			Policy mixes			
	Model 1			Model 2	Model 3	Model 4	Model 5
L.STP	0.006^***^			0.004^***^	0.002		0.004^***^
	(0.001)			(0.001)	(0.001)		(0.001)
L.DTP		0.005^***^		−0.003		0.002	−0.004
		(0.002)		(0.003)		(0.002)	(0.020)
L.ETP			0.010^***^		0.005^***^	0.009^***^	0.007^***^
			(0.002)		(0.002)	(0.001)	(0.002)
L.STP*L.DTP				0.001^***^			
				(0.000)			
L.STP*L.ETP					0.001^***^		
					(0.000)		
L.DTP*L.ETP						0.005^*^	
						(0.003)	
L.STP*L.DTP*L.ETP							0.097^**^
							(0.045)
Constant	−4.084^***^	−4.077^***^	−4.128^***^	−4.076^***^	−4.109^***^	−3.090^***^	−4.121^***^
	(0.162)	(0.162)	(0.161)	(0.162)	(0.161)	(0.114)	(0.162)
Year/Region/Industry	Yes	Yes	Yes	Yes	Yes	Yes	Yes
*N*	29,795	29,795	29,795	29,795	29,795	29,795	29,795
Adj-*R*^2^	0.146	0.146	0.147	0.147	0.148	0.142	0.148
*F*-value	166.217	159.575	168.192	133.613	138.473	242.390	126.063

#### 4.3.2. Replacement of the dependent variable

In this section, this study replaces the dependent variable with the number of patents applied. Regressions are performed again. From the results in Table 9, we find that all the coefficients of different types of talent policies and talent policy mixes on corporate innovation are consistent with the baseline one. The results remain stable.

**Table 9:** Estimation for substituting the dependent variables.

Variables	Single policy			Policy mixes			
	Model 1			Model 2	Model 3	Model 4	Model 5
STP	0.007^***^			0.005^***^	0.003^*^		0.005^***^
	(0.001)			(0.001)	(0.001)		(0.001)
DTP		0.006^***^		−0.004		0.003^*^	0.001
		(0.002)		(0.003)		(0.002)	(0.002)
ETP			0.012^***^		0.007^***^	0.011^***^	0.010^***^
			(0.002)		(0.002)	(0.001)	(0.002)
STP* DTP				0.001*^**^			
				(0.000)			
STP* ETP					0.001^***^		
					(0.000)		
DTP* ETP						0.004^*^	
						(0.002)	
STP* DTP* ETP							0.056^*^
							(0.032)
Constant	−4.211^***^	−4.202^***^	−4.267^***^	−4.203^***^	−4.246^***^	−2.861^***^	−3.837^***^
	(0.159)	(0.159)	(0.159)	(0.159)	(0.159)	(0.106)	(0.132)
Year/Region/Industry	Yes	Yes	Yes	Yes	Yes	Yes	Yes
*N*	33,441	33,441	33,441	33,441	33,441	33,441	33,441
Adj-*R*^2^	0.138	0.137	0.139	0.138	0.140	0.128	0.135
*F*-value	190.202	181.381	191.659	152.694	158.066	251.590	259.423

#### 4.3.3. Alteration of the regression method

Since the minimum value of observed patent data is 0, referring to the study of Zhang D. et al. (2022), we use the panel Tobit model to avoid the bias in the estimations brought by patent data with a constant positive value. When running the panel Tobit model, the lower bound is set to 0. The results are represented in Table 10. The conclusions are still consistent.

**Table 10:** Estimation for altering the regression method.

Variables	Single policy		Policy mixes
	Model 1			Model 2	Model 3	Model 4	Model 5
STP	0.202^***^			0.077^***^	0.076^*^		0.112^***^
	(0.032)			(0.014)	(0.040)		(0.035)
DTP		0.192^***^		−0.026		0.020	0.009
		(0.050)		(0.043)		(0.019)	(0.058)
ETP			0.380^***^		0.283^***^	0.130^***^	0.315^***^
			(0.040)		(0.063)	(0.016)	(0.043)
STP* DTP				0.006^*^			
				(0.003)			
STP*ETP					0.017^***^		
					(0.006)		
DTP*ETP						0.005^*^	
						(0.003)	
STP*DTP* ETP							1.637^*^
							(0.910)
Constant	−166.859^***^	−166.541^***^	−169.849^***^	−52.975^***^	−169.041^***^	−45.324^***^	−169.548^***^
	(6.367)	(6.356)	(6.386)	(2.127)	(6.384)	(1.641)	(6.389)
Year/Region/Industry	Yes	Yes	Yes	Yes	Yes	Yes	Yes
*N*	33,441	33,441	33,441	33,441	33,441	33,441	33,441

## 5. Discussion

This paper first empirically demonstrates the effectiveness of supply-side policy, environmental-side policy and demand-side policy instruments on corporate innovation, thereby supporting H1. Based on the large sample including 33,441 observations, we contribute to extant talent policy literature by moving beyond the perspective of single talent policy (e.g., Toma and Villares-Varela, 2019; Xu et al., 2022). Through classifying talent policy instruments (i.e., STP, DTP, and ETP), the research findings add our understanding of the talent policy instruments in a broad manner. We can conclude that it is possible for government use all kinds of talent policies to improve corporate innovation.

Second, this study uncovers the synergistic effects of talent policy mixes, thereby supporting H2 and answering the call for more research on policy mixes (Weber and Rohracher, 2012). Iglesias et al. (2011) believed that policy synergy was advantageous for increasing the effectiveness of policies and achieving Pareto optimum. Indeed, our results suggest that different types of talent policy mixes can all positively affect corporate innovation. Furthermore, compared with the impact caused by two types of talent policy mixes, the positive impact of STP-DTP-ETP mixes on corporate innovation is greater. This corroborates the idea of Guerzoni and Raiteri (2015) that a balance of policies should be the best choice. In addition, the Patent Law of the People’s Republic of China divides patents into the categories of invention, utility, and design (Tan et al., 2021). We thereby conduct statistics on the distribution of patent types in our sample and find that the percentage of invention patents is 28.367% and the percentage of utility and design patents is 71.633%. We concurrently conduct regressions by using invention patents granted, and the utility and design patents granted as dependent variable. The results show that the effects of different types of talent policies and their mixes are not changed (see Appendix A in Supplementary material), which reflects that specific types of patents will not affect the conclusion of this paper.

Finally, this paper introduces a relatively rare variable, named talent gathering, in the interaction between government and enterprises to verify H3. We expose nonlinear relationship between STP-DTP-ETP mixes and corporate innovation. Specifically, as the talent gathering reach a certain level, the STP-DTP-ETP mixes have a greater positive impact on corporate innovation. This paper deepens and expands the study of Chen and Wang (2022) by revealing a greater impact not only a positive impact after the number of talent reaches a certain level.

## 6. Conclusion and implications

### 6.1. Conclusion

By using the sample of China’s listed companies during the period 2007–2020, this paper adopts the multidimensional fixed-effect OLS to explore the impact of different types of talent policies and talent policy mixes on corporate innovation, and employs threshold regression model to detect the threshold effect of talent gathering in the framework of government-enterprise interaction. The results are shown as follows: First, the supply-side talent policy (STP), demand-side talent policy (DTP), and environmental-side talent policy (ETP) all positively affect corporate innovation. Companies that receive different types of talent policies tend to have high innovative performance. Second, talent policy mixes have a significant synergy on corporate innovation. STP-DTP mixes, STP-ETP mixes, DTP-ETP mixes, and STP-DTP-ETP mixes significantly promote corporate innovation. And the effect of STP-DTP- ETP mixes is greater than that of any two types of talent policy mixes. Finally, talent gathering has a threshold effect on the relationship between STP-DTP-ETP mixes and corporate innovation. When talent gathering has a low level, the effect of STP-DTP-ETP mixes on corporate innovation is small. The STP-DTP-ETP mixes have a greater positive impact when talent gathering reach a certain level.

### 6.2. Policy implications

We propose the following recommendations in light of the aforementioned research findings. First, the government is recommended to increase talent policy efforts in supporting the enterprises’ innovation and introduce a series of more diverse talent policies, resulting in better use of the role of different types of talent policies in the incentive effect on enterprises. Second, when designing and formulating talent policies, the policymakers should value both improving a single talent policy and combining different types of talent policies. That is, attention should be paid to the coordination of supply-side, demand-side and environment-side talent policies in order to maximize the synergistic effect of talent policies. The “combination boxing” issued by the talent policies can effectively promote the corporate innovation. Finally, the government should create a good environment for enterprises to gather talent, such as improve the market management system and develop a scientific and orderly factor market system. Companies should set up effective systems for training and promoting employees, and employ individuals with a variety of skills, so as to better leverage the different types of talent policies.

### 6.3. Limitation and future research

This paper has certain limitations. First, this research uses the number of patents to reflect corporate innovation. The activity of obtaining patents may not always represent innovative outcomes at the corporate level (Sampson, 2007). We encourage follow-up studies to examine other forms of corporate innovation, such as new product output value, new product acceptance, technology shareholding, etc. Second, our study focuses on Chinese A-share listed firms. Arguably, the sample data suit the demands of this paper. Future research in other countries could be needed to test how far our findings can be generalized. Finally, we only focus on the threshold effect of talent gathering between talent policies mixes and corporate innovation. In terms of technological innovation processes, digitization is giving an increasing number of possibilities for businesses (Secundo et al., 2020). Digitization has a significant impact on entrepreneurial products and procedures. Thus, we encourage future research to consider digital transition synchronously. Despite the aforementioned limitations, we hope that our study could encourage more investigation into the relationship between different types of talent policies and their mixes and corporate innovation.

## Data availability statement

The raw data supporting the conclusions of this article will be made available by the authors, without undue reservation.

## Author contributions

QC: implement the work, writing, evaluation, discussion, and proofreading. TS: framing the paper, study design, data provider, discussion, and writing. TW: writing, evaluation, discussion, and proofreading. All authors contributed to the article and approved the submitted version.

## Conflict of interest

The authors declare that the research was conducted in the absence of any commercial or financial relationships that could be construed as a potential conflict of interest.

## Publisher’s note

All claims expressed in this article are solely those of the authors and do not necessarily represent those of their affiliated organizations, or those of the publisher, the editors and the reviewers. Any product that may be evaluated in this article, or claim that may be made by its manufacturer, is not guaranteed or endorsed by the publisher.

## Supplementary material

The Supplementary material for this article can be found online at: https://www.frontiersin.org/articles/10.3389/fpsyg.2022.1069776/full#supplementary-material
