# Supplementary material for: Synergy effect of talent policies on corporate innovation—Evidence from China
Source: Front Psychol. 2023 Jan 18;13:1069776. doi: 10.3389/fpsyg.2022.1069776 (PMC9889546; doi:10.3389/fpsyg.2022.1069776)
Supplement: Supplementary file 1 [file Table_1.DOCX]

Appendix A. Estimation for different patent types.

| Variables | Single policy | | | Policy mixes | | | | |
| --- | --- | --- | --- | --- | --- | --- | --- | --- |
|  | Model 1 | | | | Model 2 | Model 3 | Model 4 | Model 5 |
| *Panel A: invention patents granted as dependent variable* | | | | | | | | |
| STP | 0.002^**^ |  |  | | 0.004 | -0.001 |  | 0.002 |
|  | (0.001) |  |  | | (0.008) | (0.001) |  | (0.007) |
| DTP |  | 0.003^**^ |  | | -0.002 |  | -0.001 | -0.001 |
|  |  | (0.001) |  | | (0.002) |  | (0.001) | (0.001) |
| ETP |  |  | 0.005^***^ | |  | 0.002 | 0.004^***^ | 0.004^***^ |
|  |  |  | (0.001) | |  | (0.002) | (0.001) | (0.001) |
| STP* DTP |  |  |  | | 0.001^***^ |  |  |  |
|  |  |  |  | | (0.000) |  |  |  |
| STP* ETP |  |  |  | |  | 0.001^***^ |  |  |
|  |  |  |  | |  | (0.000) |  |  |
| DTP* ETP |  |  |  | |  |  | 0.001^**^ |  |
|  |  |  |  | |  |  | (0.000) |  |
| STP* DTP* ETP |  |  |  | |  |  |  | 0.108^***^ |
|  |  |  |  | |  |  |  | (0.030) |
| Constant | -2.683^***^ | -2.681^***^ | -2.709^***^ | | -2.679^***^ | -2.692^***^ | -2.706^***^ | -2.704^***^ |
|  | (0.110) | (0.111) | (0.110) | | (0.087) | (0.277) | (0.110) | (0.110) |
| Year/Region/Industry | Yes | Yes | Yes | | Yes | Yes | Yes | Yes |
| N | 33441 | 33441 | 33441 | | 33441 | 33441 | 33441 | 33441 |
| Adj-R^2^ | 33,441 | 33,441 | 33,441 | | 33,441 | 33,441 | 33,441 | 33,441 |
| F-value | 0.119 | 0.119 | 0.120 | | 0.120 | 0.121 | 0.120 | 0.121 |
| *Panel B: utility and design patents granted as dependent variable* | | | | | | | | |
| STP | 0.002** |  |  | | 0.002* | -0.007 |  | 0.001 |
|  | (0.001) |  |  | | (0.001) | (0.011) |  | (0.001) |
| DTP |  | 0.006*** |  | | -0.003 |  | -0.002 | -0.001 |
|  |  | (0.001) |  | | (0.002) |  | (0.002) | (0.002) |
| ETP |  |  | 0.005*** | |  | 0.002 | 0.005*** | 0.004*** |
|  |  |  | (0.001) | |  | (0.001) | (0.001) | (0.001) |
| STP* DTP |  |  |  | | 0.001** |  |  |  |
|  |  |  |  | | (0.000) |  |  |  |
| STP* ETP |  |  |  | |  | 0.001*** |  |  |
|  |  |  |  | |  | (0.000) |  |  |
| DTP* ETP |  |  |  | |  |  | 0.005* |  |
|  |  |  |  | |  |  | (0.003) |  |
| STP* DTP* ETP |  |  |  | |  |  |  | 0.056** |
|  |  |  |  | |  |  |  | (0.027) |
| Constant | -2.898*** | -2.605*** | -2.925*** | | -2.894*** | -2.909*** | -3.275*** | -2.922*** |
|  | (0.134) | (0.111) | (0.133) | | (0.134) | (0.133) | (0.081) | (0.111) |
| Year/Region/Industry | Yes | Yes | Yes | | Yes | Yes | Yes | Yes |
| N | 33,441 | 33,441 | 33,441 | | 33,441 | 33,441 | 33,441 | 33,441 |
| Adj-R^2^ | 0.117 | 0.068 | 0.117 | | 0.117 | 0.117 | 0.112 | 0.117 |
| F-value | 122.578 | 222.055 | 125.749 | | 98.217 | 102.067 | 234.576 | 194.493 |
